# A Case of Familial Mediterranean Fever Diagnosed After Oral Administration of a Gonadotropin-Releasing Hormone (GnRH) Antagonist

**DOI:** 10.7759/cureus.96611

**Published:** 2025-11-11

**Authors:** Hikari Ueno, Rena Ohara, Sari Nakao, Junichi Sasaki, Toyomi Satoh

**Affiliations:** 1 Obstetrics and Gynaecology, University of Tsukuba Hospital, Tsukuba, JPN; 2 Perinatology, Moriya Daiichi General Hospital, Moriya, JPN; 3 Obstetrics and Gynaecology, University of Tsukuba, Tsukuba, JPN; 4 Obstetrics and Gynaecology, Moriya Daiichi General Hospital, Moriya, JPN

**Keywords:** cyclic peritonitis, familial mediterranean fever, gnrh antagonist, gynecological disorder, menstruation

## Abstract

Familial Mediterranean fever (FMF) is a juvenile-onset autoinflammatory disease involving recurrent peritonitis. Although the pathogenesis of the disease remains unclear, FMF is caused by dysfunction of the pyrin protein, which suppresses the activity of the inflammasome, a component of the inflammatory pathway, and various factors have been identified. In particular, the high frequency of female patients suggests an association with the menstrual cycle. We describe a case of peritonitis that developed in association with menstruation but was initially treated as a gynecological symptom due to the presence of uterine fibroids. The diagnosis of FMF was made after the use of gonadotropin-releasing hormone (GnRH) antagonists to eliminate the association with menstruation. This case emphasizes the potential for initial misdiagnosis due to the presence of an organic disease, particularly in FMF, and highlights the importance of considering both clinical findings and the disease course.

## Introduction

Familial Mediterranean fever (FMF) is a genetic disorder characterized by the sudden onset of periodic fever, serositis, arthritis, and rashes; symptoms often persist for several days and then resolve spontaneously [1,2]. The disease concept was established in the Mediterranean coastal region, and the responsible gene and its product, pyrin, were identified after 1997. Pyrin is one of the innate immune sensors that detect specific pathogens or toxins that have invaded the body, forming a large protein complex called an inflammasome. When the pyrin inflammasome detects a pathogen, it activates and triggers an inflammatory response to eliminate the pathogen. It is assumed that FMF develops when a mutation occurs in the MEFV gene, causing its function to become abnormal. This disease is generally considered to be autosomal recessive, but there are reports of familial cases exhibiting an autosomal dominant inheritance pattern, as well as cases without detectable genetic mutations. Approximately 80% of patients with FMF are symptomatic before 20 years of age; it is rare for the disease to occur after the age of 40, and these cases are often mild [2]. The interval between the onset of symptoms is usually every four weeks. Blood tests show elevated levels of WBCs and CRP, and abnormally high levels of serum amyloid A. Long-term FMF can cause organ amyloidosis.

We report a case of a Japanese woman with multiple uterine fibroids who developed periodic abdominal pain and fever and was diagnosed with FMF after pseudo-menopausal therapy with an oral gonadotropin-releasing hormone (GnRH) antagonist. This case had several important features, including the age at onset (over 40 years), which is unusual for FMF. We believe that this case is very instructive for obstetricians and gynaecologists when establishing a diagnosis in patients with organic gynaecological diseases. The patient provided consent for the publication of this report.

## Case presentation

**Figure 1:**
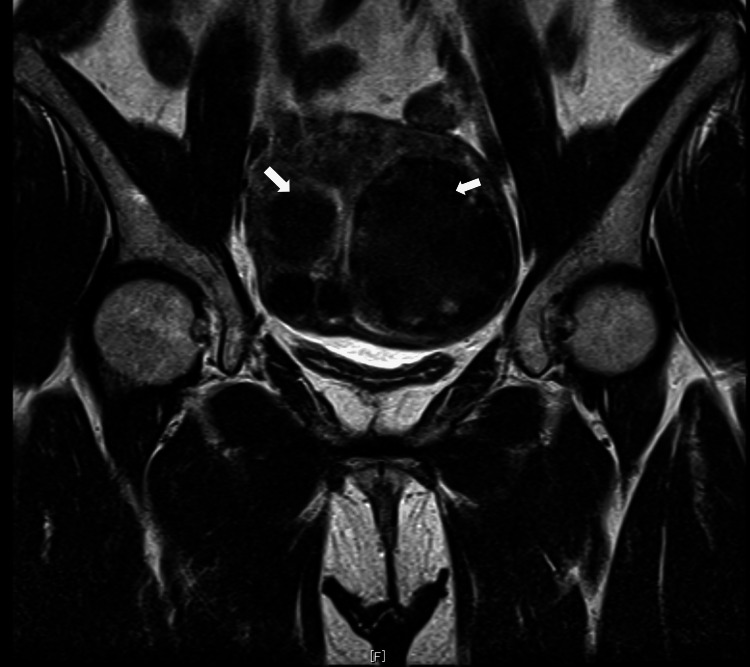
MRI (T2-weighted image, coronal section). Only multiple uterine fibroids are visible.

This case involved a 46-year-old woman. She had a history of cesarean delivery at 36 years of age because of non-reassuring fetal status during labor. She had multiple fibroids and had been followed up regularly at our hospital after delivery. Her family history was unremarkable except for suspected Sjögren's syndrome in her mother. Her menstrual cycle was 26-28 days. Although her menstrual flow was moderate for the first 2-3 days, her anemia was well controlled by oral iron therapy, and she had no desire for surgery. Approximately eight years after delivery (at 44 years of age), she began to experience abdominal pain on the third or fourth day of menstruation accompanied by fever (above 38 °C). These symptoms began to recur every three months. When symptoms appeared, both WBC (maximum 16,000/μl) and CRP (maximum 9.3 mg/dl) levels were elevated, and symptoms of peritoneal irritation and paralytic ileus were observed. Symptoms resolved within three days. Although fever is atypical, given that the patient also had multiple fibroids, and considering the possibility that uterine fibroids could be the main cause of the abdominal pain due to high menstrual volume, menstrual control was considered necessary for peritonitis associated with gynecological disease. Treatment with dienogest, a type of progestin used to treat endometriosis and adenomyosis (known by the brand name Dinagest and usually abbreviated as DNG in Japan), and Chinese herbal medicine was initiated, but both were discontinued owing to side effects and poor efficacy, as reflected in the repeated recurrence of symptoms during treatment. Computed tomography, colonoscopy, and bacteriological examinations for Chlamydia and gonorrhea were performed; however, no cause could be identified. The patient was referred to Tsukuba University for a full MRI scan, and no lesions other than known uterine fibroids were noted (Figure 1). Accordingly, treatment with a GnRH antagonist for pseudo-menopause was initiated to monitor her progress, considering the possibility of non-visible endometriosis lesions. Since symptoms appeared with menstruation, it was thought that stopping menstruation might suppress them. One month after being administered the GnRH antagonist, she came to our hospital for an evaluation of side effects. No menstrual bleeding was observed; however, she developed acute peritonitis approximately 32 days after the menstrual period preceding the start of treatment. If she had menstruated, it would have been around the third or fourth day, with elevated WBC and CRP levels. She did not receive any treatment owing to the delayed timing of her visit, and symptoms resolved spontaneously. This episode suggested that periodic peritonitis may have occurred independently of menstruation and regardless of the presence of uterine fibroids. The absence of menstrual bleeding during the symptom onset cycle, which coincided with the third to fourth day of menstruation (if present), along with the spontaneous resolution of fever within a few days without treatment, strongly suggested FMF.

After consultation with physicians specializing in rheumatology, allergy, and collagen diseases, blood tests were performed to rule out collagen diseases, primarily rheumatoid arthritis and other inflammatory disorders, and all results were negative (Table 1).

**Table 1:** List of laboratory test results for differential diagnosis. ANA: Anti-nuclear antibody; RF: Rheumatoid factor; T-SPOT.TB: Tuberculosis enzyme-linked immunospot assay; β-D-glucan: Beta-D-glucan (fungal cell wall component test); C3: Complement component 3; C4: Complement component 4; CH50: Total hemolytic complement activity; IgA: Immunoglobulin A; IgM: Immunoglobulin M; IgG: Immunoglobulin G.

Test	Result	Reference Range
Anti-DNA antibody	Negative	-
Anti-nuclear antibody (ANA)	Negative	-
Anti-SSA antibody	Negative	-
Anti-SSB antibody	Negative	-
Rheumatoid factor (RF)	Negative	-
T-SPOT.TB	Negative	-
β-D-glucan	Negative	-
Complement C3	123 mg/dL	65-135 mg/dL
Complement C4	30 mg/dL	10-35 mg/dL
CH50	58.9 U/mL	31.6-57.6 U/mL
IgA	192 mg/dL	110-410 mg/dL
IgM	103 mg/dL	46-260 mg/dL
IgG	1,207 mg/dL	870-1,700 mg/dL

**Figure 2:**
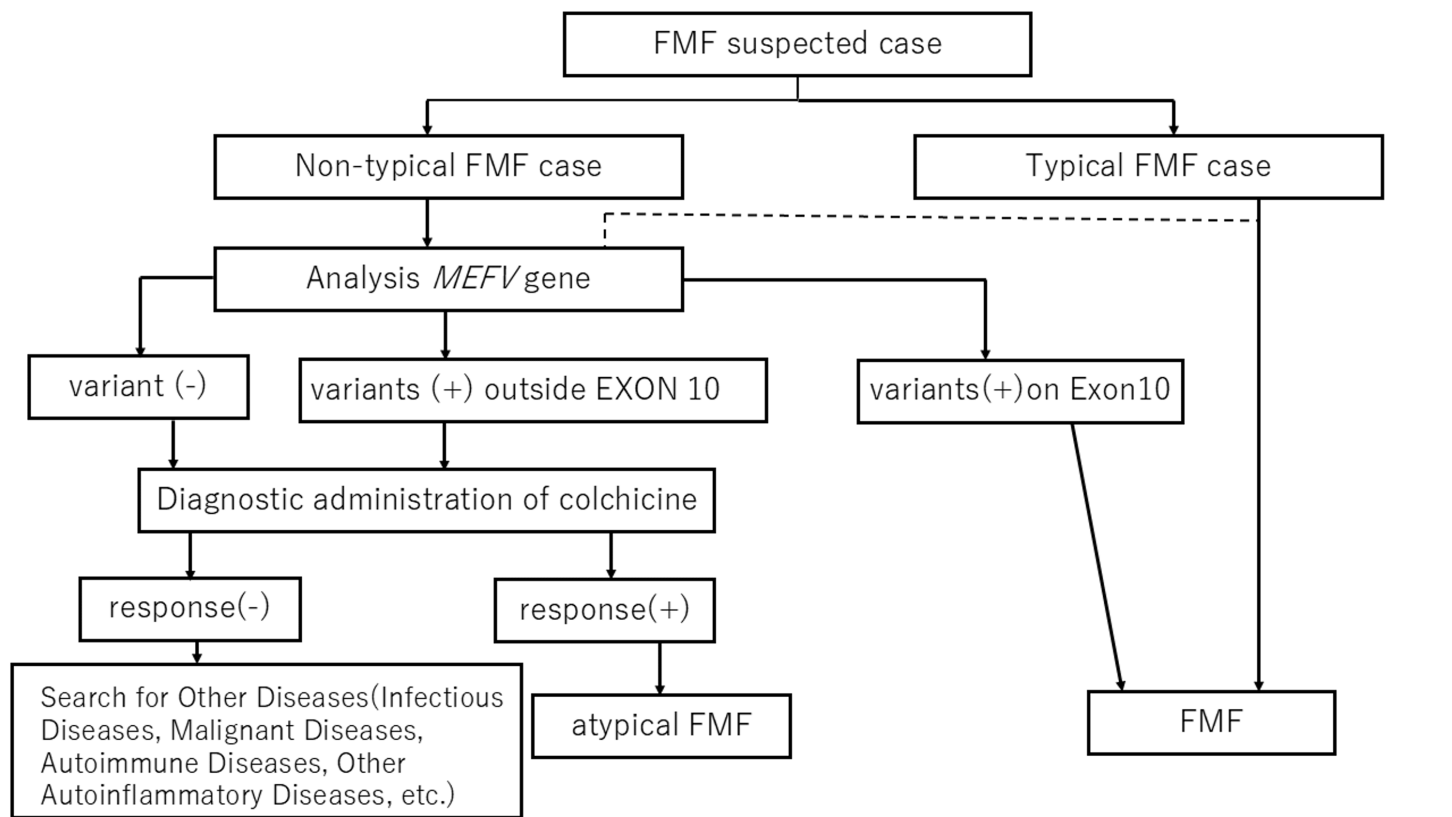
Japanese criteria for the diagnosis of familial Mediterranean fever (FMF). Essential Criteria: Three or more episodes of fever ≥38 °C lasting 12-72 hours. During fever, there is a marked elevation of inflammatory markers such as CRP and serum amyloid A (SAA). These markers return to normal during the intercritical period. Supplementary Criteria: (1) Presence of any of the following accompanying symptoms during fever: (a) Abdominal pain due to non-localized peritonitis; (b) Thoracic or back pain due to pleurisy; (c) Arthritis; (d) Pericarditis; and (e) Testicular serositis. (2) Attacks disappear or are reduced with prophylactic oral colchicine therapy. Cases meeting at least one major criterion and one or more minor criteria are clinically diagnosed as typical FMF. For suspected but atypical FMF, the diagnostic flowchart should be followed.

It was therefore considered likely that FMF was the cause, based on the typical symptoms of fever and abdominal pain attacks occurring within one week and the absence of other diseases. The patient was started on colchicine while continuing GnRH antagonist treatment, and peritonitis did not develop during the next expected menstrual cycle. Thereafter, she continued taking GnRH antagonists and colchicine, with dose adjustment for six months. She has been taking colchicine without symptom recurrence and is currently continuing GnRH antagonist therapy. After using a GnRH antagonist for six months, she switched to DNG to control menstruation, which resumed promptly without peritonitis associated with the menstrual cycle, unlike during the previous treatment attempt. Based on the effectiveness of colchicine and the presence of typical symptoms, she was diagnosed as a typical case of FMF in accordance with Japan’s diagnostic criteria (Figure 2).

The patient does not wish to undergo genetic testing at present. Based on a detailed family history, her brother also experienced periodic fever and abdominal pain for several years and had been treated for a psychosomatic disorder of unknown cause after repeated visits to various medical departments.

## Discussion

In Japan, a nationwide survey was conducted in 2009 to elucidate the pathophysiology of FMF and establish treatment guidelines. Analysis revealed that the age of onset in Japan was 18.2 ± 14.3 years, higher than in overseas cases, and that it took an average of 8.8 years from onset to diagnosis. Internationally, FMF is typically diagnosed using the Tel-Hashomer criteria; however, in Japan, diagnosis is based on criteria reflecting the clinical features observed in the Japanese population (Figure 2). This is because the genetic mutations observed in typical FMF patients in Japan predominantly involve exon 10, whereas atypical cases frequently exhibit mutations outside exon 10. These mutations have been reported as genetic polymorphisms with low penetrance, meaning they are carried at a higher rate by healthy individuals in Japan compared to other countries. Therefore, FMF diagnosis based solely on genetic mutations is not suitable in Japan. As guided by the European Alliance of Associations for Rheumatology (EULAR) recommendations, diagnosis is primarily based on clinical findings, and genetic testing for confirmation is not mandatory. Diagnosis should be confirmed by a physician experienced in treating FMF [3,4,5]. FMF is slightly more common in women than in men, with a reported ratio of 1:1.3 (men:women) in Japan [6]. The number of FMF cases has been increasing in recent years, with approximately 500 cases diagnosed annually in Japan. As mentioned above, patients with FMF are typically young; however, in Japan, FMF has been reported in women aged 30-40 years. Approximately 40% of Japanese FMF patients present with atypical symptoms (e.g., fever lasting only a few hours, absence of fever exceeding 38 °C, mild abdominal pain without peritoneal irritation, and muscle pain). Compared to cases overseas, the incidence of amyloidosis appears to be lower [1,2,6]. In the present case, FMF was observed in a 46-year-old woman.

The pathogenesis of FMF has not been fully elucidated. Although MEFV is a disease-related gene located on the short arm of chromosome 16, its penetrance is not high. There are many cases with typical symptoms in which MEFV abnormalities are absent, suggesting that other factors are involved in its pathogenesis. FMF is believed to be triggered by psychological stress, overwork, and other factors, and menstruation has been identified as a trigger in women [3,4,7-10]. For cases triggered by menstruation, it has been speculated that hormonal fluctuations during menstruation are responsible; however, the mechanism has not been fully elucidated and remains a matter of conjecture.

In our case, the patient had a history of multiple fibroids and associated excessive menstruation. The symptoms of FMF were typical, suggesting that diagnosis should have been straightforward. However, the factors that made the diagnosis difficult were as follows: (1) obstetricians and gynecologists were not familiar with FMF; (2) the patient had a gynecological disorder involving symptoms of dysmenorrhea; (3) symptoms did not occur during every menstrual cycle but every two to three months; and (4) her age was over forty. Therefore, it was assumed that the symptoms accompanying menstruation were caused by myoma, and the duration from the onset of symptoms to diagnosis was approximately three years.

In previous literature, Lidar M et al. reported that the diagnosis of FMF was significantly delayed in females, immigrants, and those with certain gene mutation patterns. The authors posited that the reasons for this delay were physician unawareness and patients’ lack of knowledge. Moreover, the diagnosis was often made only after changes in disease pattern were recognized [11]. In the present study, the use of a GnRH antagonist and pseudo-menopause therapy made it possible to investigate the possibility of a non-gynecological disease. In addition, because the patient had undergone a thorough examination for other medical conditions when symptoms were observed prior to diagnosis, the differential diagnosis was possible, contributing to a smooth diagnosis once FMF was considered. In this case, the patient achieved a complete pseudo-perimenopausal state with the use of a GnRH antagonist. It is difficult to speculate about the best hormonal therapy to control menstruation based on this single case.

This case also raised several questions about the relationship between FMF and menstruation. In many cases of FMF reported in Japan, menstruation has been identified as a trigger [3,4,7-10]. The fact that FMF often occurs every four weeks, consistent with the duration of a typical menstrual cycle, supports a potential link between the onset of FMF and menstruation. The large number of reports indicating an association between menstruation and FMF in Japan has been attributed to differences in causative genes between Japan and other countries [10]. However, considering that FMF symptoms occurred even when pseudo-menopause therapy or dienogest (DNG) therapy, both of which are thought to be effective for FMF since they suppress menstruation, was administered in this case, the relationship may not be directly related to menstruation or to the decrease in hormone levels during the menstrual cycle [7]. Further investigation is needed to determine whether these factors (i.e., menstruation or decreased hormone levels) are indeed triggers. It is also possible that the higher number of reports of menstruation-related symptoms in Japan compared with other countries is related to differences in menstrual management practices. In Japan, the distribution of oral contraceptives was slower than in other countries because of concerns about the spread of sexually transmitted infections or the risk of pulmonary embolism. The percentage of women receiving hormone therapy is far lower in Japan than elsewhere. Consequently, many women have spontaneous menstrual cycles [12,13], which may explain the high number of FMF cases coinciding with menstruation in Japan.

## Conclusions

In the present study, we encountered a case of FMF accompanied by a gynecological organic disease. Symptoms occurring during menstruation were initially considered to be caused by the organic disease; however, a diagnosis was established through pseudo-menopause therapy with a GnRH antagonist. This case suggests that there is room for a multifaceted investigation. In any case, it is essential to listen sincerely to the patient’s complaints and to carefully consider all possible diseases without being misled by the presence of an organic disorder. Clinicians should also seek to deepen their understanding of pathologies that may be influenced by physiological factors, such as the female reproductive cycle and hormonal changes, even if these lie outside their primary field of expertise.
